# More than Nutrition: Therapeutic Potential of Breast Milk-Derived Exosomes in Cancer

**DOI:** 10.3390/ijms21197327

**Published:** 2020-10-03

**Authors:** Ki-Uk Kim, Wan-Hoon Kim, Chi Hwan Jeong, Dae Yong Yi, Hyeyoung Min

**Affiliations:** 1 College of Pharmacy, Chung-Ang University, Seoul 06974, Korea; hakiukah@cau.ac.kr (K.-U.K.); vessel37@cau.ac.kr (W.-H.K.); tolast12@cau.ac.kr (C.H.J.); 2 Department of Pediatrics, Chung-Ang University College of Medicine, Seoul 06974, Korea; 3 Department of Pediatrics, Chung-Ang University Hospital, Seoul 06973, Korea

**Keywords:** human milk, nutrient, microbiota, exosomes, microRNAs, cancer

## Abstract

Human breast milk (HBM) is an irreplaceable source of nutrition for early infant growth and development. Breast-fed children are known to have a low prevalence and reduced risk of various diseases, such as necrotizing enterocolitis, gastroenteritis, acute lymphocytic leukemia, and acute myeloid leukemia. In recent years, HBM has been found to contain a microbiome, extracellular vesicles or exosomes, and microRNAs, as well as nutritional components and non-nutritional proteins, including immunoregulatory proteins, hormones, and growth factors. Especially, the milk-derived exosomes exert various physiological and therapeutic function in cell proliferation, inflammation, immunomodulation, and cancer, which are mainly attributed to their cargo molecules such as proteins and microRNAs. The exosomal miRNAs are protected from enzymatic digestion and acidic conditions, and play a critical role in immune regulation and cancer. In addition, the milk-derived exosomes are developed as drug carriers for delivering small molecules and siRNA to tumor sites. In this review, we examined the various components of HBM and their therapeutic potential, in particular of exosomes and microRNAs, towards cancer.

## 1. Introduction

Human breast milk (HBM) is an irreplaceable source of nutrition for early infant growth and development. For this reason, the World Health Organization and the American Academy of Pediatrics recommend exclusive breastfeeding for at least 6 months and to continue breastfeeding until the age of 2 years [1,2,3]. It is widely known that HBM provides advantages for cognition and development in the short and long term [3,4]. Breast-fed children are known to have a low prevalence of necrotizing enterocolitis (NEC), gastroenteritis, otitis media, respiratory diseases, and acute diseases, as well as obesity, inflammatory bowel disease, and diabetes [3,5,6]. In addition, breastfeeding for more than 6 months reduces the risk of acute lymphocytic leukemia and acute myeloid leukemia, and there is evidence of reduced morbidity associated with lymphoma and other tumors [3,5,6,7]. Furthermore, breastfeeding mothers show various short-term benefits and long-term positive effects on cardiovascular disease, diabetes, and bone density [5]. In particular, breastfeeding is known to lower the risk of breast and ovarian cancer.

Although various multinational companies are conducting research to develop breast milk replacements, it has not yet been possible to completely replace breast milk by any method [8]. This is because HBM contains various ingredients that have not yet been identified, so it is difficult to artificially reproduce them. HBM contains not only nutritional components, such as macronutrients and micronutrients, but also various non-nutritional proteins or cellular components, including immune components, hormones, and growth factors [9,10,11]. In recent years, studies on HBM components, such as human milk oligosaccharides (HMOs) and fatty acids, have been actively conducted. The HBM components that have not been well appreciated in the past, such as the microbiome, extracellular vesicles (EVs) or exosomes, and microRNAs (miRNAs), are being examined, assisted by the development of various testing techniques [11,12,13]. Some of these components consistently show no considerable differences between regions and races, such as protein components and with regard to the energy content. However, there are differences according to the diet or weight of the breastfeeding mother in components such as vitamin A, vitamin D, water-soluble vitamins, and the composition of fatty acids, and also in other components according to the underlying condition of the breastfeeding mother [3,11,12]. In addition, the maturation, colonization, and immunity acquisition of immature intestinal mucosa are obtained through breastfeeding, and the incidence of various diseases is lower in breastfed infants than that in formula-fed infants. Considering these aspects, the HBM components, such as the immune components and exosomes, would be helpful in predicting and treating diseases in actual clinical practice.

In this article, we examined the various well-known components of HBM and those that are currently being studied, focusing on exosomes. In addition, we described how these ingredients affect the health of children and breastfeeding mothers and especially their potential use in diseases such as cancer.

## 2. Human Breast Milk Components

### 2.1. Nutritional Components (Macronutrients and Micronutrients)

As already known, HBM is mainly composed of macronutrients such as carbohydrates, proteins, and fats, along with 87–88% water [11]. Among them, carbohydrates are the most important component in HBM and play a critical role in infant nutrition. Although lactose is a major carbohydrate constituent of HBM, HMOs, which have recently been attracting attention, are unique components of HBM and are the third largest constituent of HBM. HMOs play a prebiotic role that affect the development of intestinal colonization and gut microbiota immediately after birth, directly affecting immunity, and also play a role in the production of short chain fatty acids [14,15]. The proteins in HBM are composed of various peptides along with a mixture of casein and whey. These proteins play an essential role in growth and development by being involved in the functionalization and organization of cells in the human body [11]. As HBM is known to play an essential role in the development of the early human immune system, whey proteins, such as alpha-lactalbumin, lactoferrin, and secretory IgA, play an important role in the immune system and have antibacterial properties [10,16,17]. Fat accounts for 50% of the total nutrition supplied by HBM, is the second largest macronutrient, and plays an important role in the development of the nervous system [18]. Fat in HBM varies according to the maternal diet and body weight during pregnancy, so there are large regional differences [18].

In addition, HBM contains various vitamins and micronutrients, such as iron, calcium, zinc, and copper. Although most of these micronutrients are sufficiently contained in breast milk, vitamin D or vitamin K may require supplementation if exclusively breastfeeding due to insufficiencies [19,20]. The nutrient content of HBM varies depending on whether it is colostrum or mature milk, and the ratio changes as lactation progresses. In addition, HBM fed to premature infants may have different components from that fed to mature infants [11].

### 2.2. Non-Nutritional Components and Clinical Applications

In addition to the nutrient components, HBM contains various non-nutritional components, bioactive proteins, and peptides. HBM is known to contain various hormones, such as parathyroid hormone, insulin, and leptin, but their functions are not fully understood [11]. However, the roles of many growth factors are well known. Epidermal growth factors play a crucial role in the maturation and healing of intestinal mucosa, and neuronal growth factors are known to be necessary for the development of the enteric nervous system and immature intestine in newborns [10,11]. Moreover, the insulin-like growth factor superfamily and vascular endothelial growth factor regulate erythropoiesis and angiogenesis, respectively [10,11].

Considering the development of the immune system through breastfeeding, the formation of a functional microbial community through colonization of microorganisms in the intestinal tract, and the low disease incidence in breast-fed infants, various components of HBM have been studied for their applicability in the clinic and infection [21]. Attempts have been made to use lactoferrin to treat colon cancer, advanced stage non-small-cell lung carcinoma, newly diagnosed lung cancer, and breast cancer in connection with regulating cellular growth and differentiation, playing an important role in immune response [22,23,24,25,26]. Another immune component, alpha-lactalbumin, is known as HAMLET (human alpha-lactalbumin made lethal to tumor cells) for its clinical applicability in oncologic diseases. Through the intratumoral administration of HAMLET, damage to the intact brain in human glioblastoma is minimized and tumor cell apoptosis is induced [27]. The effects of alpha-lactalbumin on apoptosis and resolution have also been confirmed in human skin papilloma and bladder cancer [28,29]. The transforming growth factor β (TGF-β) contained in milk acts on immunomodulation and cell proliferation and differentiation, particularly in pediatric Crohn’s disease, and is used for enteral nutrition [21].

### 2.3. Microbiomes and Their Derived Extracellular Vesicles

Until the 20th century, HBM was considered sterile, and bacterial species identified in HBM were believed to be due to contamination or infection. However, it was known to some researchers that HBM also contains commensal bacteria, and rich and diverse communities have been identified by new testing techniques such as Next Generation Sequencing [30,31,32]. Through these advanced techniques, *Staphylococcus* and *Streptococcus* were found to be the predominant core genera in HBM, despite differences in regions and test techniques [11]. In addition, Togo et al. confirmed the diversity of human milk microbiota through a systematic review and identified commonly found species: *Staphylococcus aureus*, *Staphylococcus epidermidis*, *Streptococcus agalactiae*, *Cutibacterium acnes*, *Enterococcus faecalis*, *Bifidobacterium breve*, *Escherichia coli*, *Streptococcus sanguinis*, *Lactobacillus gasseri,* and *Salmonella enterica* [33].

The origin of the HBM microbiota remains unknown. The possibility of contamination of the skin or oral cavity cannot yet be ruled out. In fact, Ramsay et al. confirmed milk-ejection reflux from the mouth of the infant to mammary ducts during breastfeeding through ultrasound [34]. However, an introduction of microbiota from an endogenous origin, such as glands, rather than contamination is possible, since anaerobic gut-associated microbiota has been identified in breast milk. The maternal gut microbiota may be absorbed through the maternal intestinal epithelium and present in the mammary glands [35,36]. It is thought that these HBM microbes act as prebiotics with HMOs and indirectly affect the infant gut and various extra-intestinal environments [37,38,39,40]. Through many studies conducted so far, vertical transmission of HBM microbiota has been shown to play an important role in the initial formation of infant gut microbiota. It is already known that there is a difference between the stool microbiota of the infant and adulthood periods in breast-fed and non-breast-fed individuals, and a role for HBM microbiota in human health is possible, as it is associated with a low incidence of various diseases such as infectious diseases and allergies in breast-fed infants [41,42].

In addition to these microbiomes, HBM was also found to contain EVs of bacterial origin [12]. EVs are nanometer-sized membrane vesicles that have various bioactive functions in intercellular communication. EVs were generally classified into exosomes (endocytic pathway), microvesicles (plasma membrane), and apoptotic bodies (plasma membrane) according to their cargos, biogenesis, and size [43]. Bacterial origin EVs are present in a variety of body fluids such as blood, urine, and stool, but their role in breast milk remains unknown. At the genus level, HBM bacteria and bacterial EVs show a significant difference. It is believed that bacterial origin EVs act on the host receptor to move bioactive molecules to host cells or to move the EVs themselves to host cells to play a role in infant gut colonization and immunity [12].

## 3. Breast Milk-Derived Exosomes

Exosomes are small membranous extracellular vesicles secreted by most eukaryotic cells into surrounding body fluids, such as blood, saliva, urine, cerebrospinal fluid, lymphatic fluid, and amniotic fluid [44,45,46]. These vesicles are 40~100 nm in diameter and are involved in cell–cell communication by transporting the bioactive cargo molecules, including DNAs, mRNAs, microRNAs, lipids, and proteins, derived from the cells of origin to the target cells. Exosomes are also present in breast milk, and the diverse health benefits of breastfeeding are attributed to exosomes, as well as to well-known immunoregulatory components such as lactoferrin, lactalbumin, lysozyme, and sIgA [47,48].

Exosomes were first extracted from human colostrum and breast milk and characterized in 2007 [47], and subsequent studies have reported the isolation and characterization of milk exosomes from cows, camels, buffalos, pigs, sheep, and pigeons [49,50,51,52,53,54,55]. Milk exosomes are secreted by mammary gland epithelial cells and also released from milk fat globules during lactation [56]. Higher concentrations of exosomes were detected in early milk collected at day 3–8 postpartum than in mature milk collected at 2 months, whereas no significant differences were found in the expression of marker proteins, such as the tetraspanins CD63, CD81, and the scavenger receptor CD36, between early and mature milk [57].

### 3.1. The Role of Milk Exosomes in Cell Proliferation and Inflammation

Studies have reported that milk exosomes function in regulating cell proliferation and inflammation. Hock et al. showed that rat milk-derived exosomes promote the viability and proliferation of intestinal epithelial cells and intestinal stem cell activity [58], supporting the previous observations that HBM reduces the incidence of NEC in infants [59,60,61,62]. Consistently, Martin et al. have also reported that human milk-derived exosomes protect intestinal epithelial cells from cell death upon exposure to oxidative stress [63]. Furthermore, a neonatal mouse intestinal organoid model and experimental NEC in C57BL/6 mouse pups supported the protective activities of HBM exosomes by decreasing inflammation and intestinal damage [64]. A reduction in intestinal damage has also been observed by exosomes derived from pasteurized breast milk and raw breast milk, indicating that milk exosomes are resistant to the pasteurization process. In addition, an increasing number of studies have reported the protective effects of milk exosomes in various in vitro and in vivo NEC models, confirming the critical role of exosomes in the prevention of NEC development in premature infants [65,66]. 

The anti-inflammatory effects of bovine milk-derived exosomes have also been shown in IL-1Ra(-/-) and collagen-induced arthritis mouse models [67]. When administered orally by oral gavage or drinking water, milk exosomes delay the onset of arthritis and reduce the swelling of ankle joints, cartilage depletion, and bone marrow inflammation. In addition, the circulating levels of MCP-1, IL-6, and anti-collagen IgG_2a_ declined, accompanied by a decrease in mRNA expression of T-bet (Th1) and ROR-γT (Th17) in the primary splenocytes. These results suggest the therapeutic potential of milk exosomes in the treatment of autoimmune and inflammatory diseases.

### 3.2. Immunomodulatory Function of Milk Exosomes

Breast milk exosomes also show immunomodulatory effects. The Gabrielsson group demonstrated that HBM-derived exosomes inhibit CD3-induced production of IL-2, IFN-γ, and TNF-α in peripheral blood mononuclear cells (PBMCs), while increasing the number of FoxP3^+^ CD4^+^ CD25^+^ regulatory T cells in PBMCs [47]. They also reported that MUC-1 expressed on HBM-derived exosomes binds to DC-SIGN on monocyte-derived dendritic cells (MDDCs) and blocks HIV-1 infection and viral transfer from MDDCs to CD4^+^ T cells [68]. In addition, human milk exosomes possess different phenotypes, depending upon maternal sensitization and lifestyle, and differentially influence allergy development in children [57]. For example, exosomes derived from mothers with an anthroposophic lifestyle are associated with a lower prevalence of allergic sensitization than those from non-anthroposophic mothers. Cow milk-derived exosomes upregulate CD69 expression in NK cells and IFN-γ production in NK cells and γδ T cells in the presence of IL-2 and IL-12 [69]. Furthermore, bovine milk exosomes induce proliferation of macrophages and RAW264.7 cells and protect macrophages against cisplatin-induced cell death [70]. These data suggest that milk exosomes potentially influence the immune system.

A proteomic analysis has been performed to compare the exosomal protein contents between bovine colostrum and mature milk (mid-lactation period) [71]. The results revealed that exosomes from colostrum are highly enriched in proteins implicated in the innate immune response, inflammatory response, acute-phase response, platelet activation, cell growth, and complement activation, whereas proteins implicated in transport and apoptosis are enriched in exosomes from mature milk [71]. A further proteomic analysis of bovine milk exosomes also reported that exosomal proteins map to the Kyoto Encyclopedia of Genes and Genomes (KEGG) pathways with immunological functions such as Fc gamma receptor-mediated phagocytosis, antigen processing and presentation, T cell receptor signaling, B cell receptor signaling, and NK cell-mediated cytotoxicity [72]. Proteome studies supported the immunoregulatory function of milk exosomes and suggested the importance of colostrum in immune defense and immune system development during the early period after birth.

### 3.3. Milk Exosomes and Cancer

The chemopreventive effects of breast milk have been well studied in childhood leukemia and lymphoma [73,74,75,76,77]. Martin et al. performed a systemic review and meta-analysis and showed that ever having been breast-fed is inversely associated with the incidence of acute lymphoblastic leukemia, Hodgkin’s disease, and neuroblastoma in childhood [78]. Similarly, a recent meta-analysis and systemic review reported that 14–19% of all childhood leukemia cases might be prevented by breastfeeding for 6 months or more, whereas breastfeeding for a short duration or non-breastfeeding may be associated with a slightly increased risk of acute childhood leukemia [7,79]. Given the severity and detrimental effects of these illnesses, any factor lowering the risk of childhood cancer would be useful.

Reif et al. reported that HBM-derived exosomes promote the proliferation of normal colon epithelial cells without affecting the growth of colonic cancer cells [80]. Although this study does not show the direct antitumor effects of human milk exosomes, it suggests the beneficial function of human milk exosomes through differential effects on normal cells compared to cancer cells. Bovine milk-derived exosomes have revealed their intrinsic antitumor activity by inhibiting the proliferation of various types of cancers, such as lung, prostate, colon, pancreatic, breast, and ovarian cancers [81]. Treatment of cancer cells with 50 μg/mL exosomal proteins for 72 h reduces cell growth by 8–47% as assessed by MTT assay, suggesting the benefit of exosomes as delivery vehicles for anticancer drugs. In addition, camel milk and its components have also been shown to exert antitumor effects in human hepatoma (HepG2), human breast cancer cells (MCF7), and murine hepatoma (Hepa 1c1c7) [82,83]. Camel milk inhibits cell growth and induces apoptosis by activating caspase-3 and death receptor DR4 and accumulates intracellular reactive oxygen species in HepG2 and MCF7 cells [83]. Badawy et al. demonstrated that camel milk and its exosomes inhibit the proliferation of MCF7 cells, accompanied by a decrease in MCF7 migration, as measured by a wound-healing assay [53]. In tumor-bearing rats, oral or local (in the tumor tissue) administration of camel milk and its exosomes significantly reduces tumor weight and progression by inducing apoptosis and inhibiting oxidative stress, inflammation, angiogenesis, and metastasis. Moreover, the numbers of CD4^+^, CD8^+^, and NKT1.1^+^ T cells increase in the spleen following treatment with camel milk and its exosomes. Although exosomes show better overall antitumor effects, the increase in the numbers of splenic T cells is more potent in milk-treated rats, suggesting that camel milk possesses more immune-stimulating constituents than exosomes.

In contrast, breast milk exosomes may negatively influence some type of cancers. TGF-β isolated from breast milk exosomes promotes the proliferation of breast cancer cells and epithelial–mesenchymal transition (EMT), as demonstrated by changes in the actin cytoskeletal structure and loss of E-cadherin expression [84]. Since epithelial cells acquire migratory and invasive properties during EMT, TGF-β in exosomes may lead to the transformation of normal cells and a change in breast cancer cells into a more aggressive and invasive tumor.

## 4. Breast Milk-Derived Exosomal MicroRNAs

MicroRNAs are endogenous small non-coding RNA molecules that are 19~24 bp in length [85,86,87]. miRNAs interact with the 3′ untranslated region (UTR) of target mRNAs and control gene expression post-transcriptionally. Ample studies have reported the function of miRNAs in diverse biological processes, such as proliferation, differentiation, cell cycle, and cell death [88,89], and dysregulated miRNAs are implicated in the development and progression of many human diseases [90,91]. In relation to cancer, miRNAs may function as tumor suppressor genes or oncogenes, and accordingly, aberrantly expressed miRNAs in particular types of cancer have been suggested as useful biomarkers for cancer diagnostics and therapeutic targets [92,93,94,95]

HBM is highly enriched in miRNAs, with more than 1400 identified miRNAs [96,97,98,99,100]. Breast milk miRNAs are thought to originate from the mammary epithelium and are present in the cells, skimmed fractions, and lipid fractions of breast milk [98]. More miRNAs are detected in the cell and lipid fractions of breast milk than the skimmed milk fraction, suggesting the importance of cell and lipid fractions in the analysis of breast milk miRNAs [96,100]. Exosomes are mostly present in the skimmed milk fraction, and exosomal miRNAs are often reported within breast milk miRNA if it has not been specifically mentioned that exosomes were isolated from milk before miRNA extraction. 

Studies have reported that milk exosomes protect exosomal miRNAs from enzymatic, chemical, or mechanical degradation. When exposed to acidic conditions that mimic gastric and pancreatic digestion, milk exosomes prevent the degradation of vulnerable miRNAs [101]. Commercial bovine milk also protects miRNAs from acidic environments and RNase treatment, safely delivering miRNAs into the digestive tract [102]. Subsequently, milk exosomes are taken up by intestinal epithelial cells by endocytosis and moved into systemic circulation [103,104]. In addition, studies have shown that milk exosomes containing miRNAs are absorbed into macrophages, PBMCs, and kidney cells, and regulate gene expression in the target cells [67,105]. For example, upon incubation of normal intestinal CRL 1831 cells, K562 leukemia cells, and Lim 1215 colon cancer cells with milk exosomes, the expression of miR-148a-3p, the most abundant miRNA in breast milk, is increased accompanied by a decrease in the expression of DNA methyltransferase1 (DNMT1), a target of miR-148a-3p [106]. Although the composition of breast milk and miRNAs vary depending on the maternal health and nutrition status and lactation stage [107,108], miR-148a-3p is highly expressed in breast milk and conserved across mammalian species, and other highly expressed miRNAs, such as miR-320, miR-375, and miR-99, are also frequently found in different species [106].

### 4.1. Immune-Regulating miRNAs

Exosomal miRNAs contribute to the physiological and therapeutic functions of milk exosomes. Several studies have reported that breast milk is rich in immune-regulating miRNAs, such as miR-125b, miR-146b, miR-155, miR-181a, and miR-181b [108,109,110]. These miRNAs are known to regulate B cell, T cell, and monocyte development, and control the innate immune response and cytokine production (Table 1). Zhou et al. demonstrated that immune-regulating miRNAs are abundant in milk exosomes [99]. Among the 453 pre-miRNAs detected in milk exosomes, 59 pre-miRNAs were immune related, based on annotation in the Pathway Central database. For example, miR-30b-5p promotes cellular invasion by directly targeting GalNAc transferase and immunosuppression by increasing IL-10 [111], whereas miR-182-5p promotes helper T cell-mediated immune response upon induction by IL-2 [112]. Other frequently found immune-related exosomal miRNAs are miR-148a-3p, miR-146a, miR-146b-5p, miR-200a-3p, and miR-29a-3p [99,106,113]. These immune-regulating miRNAs may be transferred into the infant body during breastfeeding and function in the development of the immune system [99]. Future studies extending beyond in silico analysis are needed to elucidate the mechanisms by which individual miRNAs control immune responses in infants.

### 4.2. Tumor-Associated miRNAs

In addition to immune-regulating miRNAs, milk exosomes also contain tumor-suppressive or oncogenic miRNAs (oncomiRs) [114]. miR-148a-3p has been shown to function as a tumor suppressor by targeting DNMT1, ERBB3, and ROCK1, which are all involved in the development, proliferation, and metastasis of tumors [106,115,116,117]. Given that miR-148a-3p is highly expressed in breast milk but less expressed in leukemia, miR-148a-3p in breast milk may be able to protect infants against childhood leukemia [79,118,119]. 

In contrast, some well-known oncomiRs, such as the miR-21, miR-155, miR-223, and miR-17-92 clusters, have been found in breast milk [120]. Of note, Melnik suggested that miRNA-21-5p, a highly expressed miRNA in human and cow milk, is one of the major environmental factors eliciting melanomagenesis through exosomal transfer of miRNAs [99,121]. miR-21-5p downregulates the expression of tumor suppressor genes, such as PTEN (phosphatase and tensin homolog), Sprouty1 and Sprouty2, and PDCD4 (programmed cell death protein 4), thereby promoting the initiation and progression of malignant melanoma [122,123,124]. It has been proposed that an exogenous supply of exosomal miR-21 through breastfeeding or milk consumption may enhance oncogenic miR-21 signaling and lead to the transition of benign melanocytes to malignant melanoma [121]. Additionally, miR-155, an oncomiR that enhances STAT3 expression by targeting SOCS1 (suppressor of cytokine signaling 1) and facilitates STAT3-mediated tumorigenesis, is also found in exosomes derived from bovine colostrum, implying the association of milk exosomal miR-155 with tumorigenesis [113,125,126]. 

Nonetheless, studies that provide a direct evidence of the role of breast milk miRNAs in the prevention or development of cancer are limited. Most studies reporting the function of milk exosomal miRNAs are in silico analyses accompanied by Gene Ontology and KEGG pathway analyses. Further investigations merging in silico analysis and in vitro and/or in vivo experimental validation are required to determine any significant association between breast milk miRNAs and cancer.

## 5. Breast Milk-Derived Exosomes as Natural Carriers for Drug Delivery

As natural carriers of endogenous biomolecules, exosomes have remarkable advantages over other drug delivery vehicles. Exosomes are biocompatible and low in toxicity and immunogenicity and have long circulating half-lives [44,45]. Furthermore, they can cross many biological barriers such as the blood–brain barrier and cytoplasmic membrane [127]. Accordingly, numerous exosome-based delivery systems have been developed for targeted drug delivery by transporting small molecules, proteins, and small interfering RNA (siRNA) to target tissues. In particular, milk exosomes possess unique benefits compared with those of other origins [128,129]. Milk exosomes can be obtained from bovine milk enabling low-cost and mass production and show cross-species tolerance [128]. Moreover, milk exosomes are stable in acidic environments and can be absorbed from the digestive tract in humans, suggesting the potential use of milk exosomes as oral delivery vehicles of drugs that are conventionally administered intravenously [130]. With additional modification for target-specific drug delivery, milk exosomes have been widely investigated as promising carriers to transport diverse biomolecules and chemotherapeutic agents.

### 5.1. Delivery of Small Molecules

Milk exosomes have been shown to deliver natural compounds isolated from plant resources, such as celastrol, anthocyanidin, and curcumin [81,131,132]. When loaded onto cow milk-derived exosomes, curcumin with poor oral bioavailability is taken up and shows enhanced antiproliferative effects compared with free curcumin in Caco-2 cells [131]. Exosomal formulation of anthocyanidin (ExoAnthos) also exerts greater antiproliferative effects than that of free Anthos in lung, breast, ovarian, colon, pancreatic, and prostate cancers in vitro, and has higher antitumor efficacy in mice bearing A549 xenografts upon oral administration [81]. In addition to the improvement in oral bioavailability, ExoAnthos is well tolerated with no systemic toxicity, as determined by biochemical and hematological parameters. Similarly, celastrol encapsulated in milk exosomes inhibits the proliferation of the non-small-cell lung carcinoma cell lines A549 and H1299 in vitro, and shows greater antitumor effects in vivo without toxicity than that of free celastrol [132].

Agrawal et al. demonstrated that paclitaxel-loaded milk exosomes (ExoPACs) inhibit the growth of human lung tumor in athymic nude mice bearing A549 xenografts [133]. The ExoPACs are highly stable in simulated gastrointestinal fluids and under cold storage conditions at −80 °C for 1 month, allowing oral delivery of paclitaxel. Furthermore, oral administration of ExoPACs maintains the number and function of immune cells with reduced systemic and tissue toxicity in mice compared with intravenous administration of the drug, indicating the safety and efficacy of drug-loaded milk exosomes. Considering the discomfort and inconvenience of the intravenous route, the use of milk exosomes would be beneficial for the oral delivery of chemotherapeutic agents with low oral bioavailability. 

### 5.2. Delivery of Nucleic Acid

Since the discovery of RNA interference mediated by siRNA, siRNAs have attracted much attention as a new generation of therapeutics based on nucleic acid. Despite the promising therapeutic potential of silencing gene expression in a sequence-specific manner, siRNAs have some intrinsic undruggable properties [134,135]. They are readily degraded by nuclease in serum and tissue and cleared by renal excretion or phagocytes in the reticuloendothelial system (RES). Negatively charged siRNAs are not easily taken up by cells due to electrostatic repulsion, and endosomal escape is required for siRNAs to reach mRNA targets, once internalized. To overcome the obstacles that limit the safe and effective application of siRNAs, the chemical modification of their nucleotide components, including ribose, phosphate, and base, as well as the special siRNA delivery systems, such as polymeric nanoparticles and liposomes, have been extensively investigated [134,135].

In addition to the oral delivery of small molecule drugs, milk exosomes have also been used for the delivery of siRNAs. siRNAs were loaded onto milk exosomes by electroporation and chemical transfection, and their gene silencing activities were tested in multiple cancers in vitro [129]. The siRNA-loaded exosomes are resistant to RNase and taken up by cancer cells accompanied by silencing of the target genes. Exo-siKRAS (siRNA against KRAS-loaded exosomes) suppresses the proliferation of A549 in vitro and exhibits antitumor effects in A549 tumor-bearing mice upon intravenous administration.

Similarly, siRNAs against *bcl-2*, an oncogene that inhibits apoptosis and promotes proliferation, were loaded onto milk exosomes using an ultrasonic approach, and their antitumor activity was tested in vitro and in vivo [136]. The results demonstrated that exosiBcl-2 (siRNA against *bcl-2*-loaded exosomes) inhibits *bcl-2* expression followed by an increase in apoptosis and a decrease in cell growth, migration, and invasion of cancer cells in vitro. An in vivo study also confirmed that intravenous administration of exosiBcl-2 suppresses tumor growth in xenograft nude mice. In view of the advantages of milk exosomes over other siRNA delivery vehicles, future studies are expected to establish optimal methods for siRNA loading, surface functionalization, and administration routes to attain effective antitumor therapies.

### 5.3. Targeted Dug Delivery

Despite excellent biocompatibility and low toxicity, exosomes from bovine milk may require further modification for targeted drug delivery. Folate receptors are highly expressed on many types of tumor cells, and accordingly, folic acid (FA)-conjugated milk exosomes have been generated for enhanced delivery of siRNAs to tumor sites [129]. FA-functionalized milk exosomes show higher accumulation in tumor tissue than non-FA milk exosomes and significantly inhibit tumor growth in vivo.

CD44, a receptor for hyaluronic acid, is overexpressed in some types of cancer such as pancreatic, lung, ovarian, and breast cancer [137]. For targeted cancer therapy, attempts have been made to generate conjugates of membrane incorporating molecule DSPE-PEG_200_ and Hyaluronan (HA), a CD44-specific ligand, which are then self-assembled into the phospholipid bilayer of milk exosomes [138]. The resulting HA-incorporated milk exosomes (HA-mExo) expose the HA ligand on the surface of the exosomes and are loaded with doxorubicin to obtain HA-mExo-Dox, a doxorubicin-loaded milk exosome with an HA ligand. The results showed that HA-mExo-Dox selectively delivers doxorubicin to CD44-overexpressing cancer cells and exerts enhanced antitumor activity.

Another example of a tumor-targeting ligand is iRGD (CRGDK/RGPD/EC), a 9-amino acid cyclic peptide that binds to ανβ3 and ανβ5 integrin on the endothelial cells of tumor vessels to promote transcytosis across tumor vasculature [139]. After translocation, the iRGD ligand is cleaved by an endogenous protease to yield a CRGDK/R peptide that serves as a ligand for the neuropilin-1 receptor to activate the transport of co-administered anticancer drugs into tumors [140]. When iRGD peptides are incorporated into milk exosomes, the oral administration of iRGD exosomes exhibits an increase in penetration and accumulation into tumors compared with intravenously administered control exosomes [130]. In contrast, the accumulation of iRGD exosomes in other organs, such as liver, heart, lung, and spleen, is decreased. Considering the increased tumor accumulation and decreased systemic distribution, iRGD exosomes might be used as promising delivery vehicles for chemotherapeutic agents with a high antitumor efficacy and decreased off-target effects. 

In addition, a milk exosome-based pH and light-sensitive drug carrier has been developed for targeting an acidic and hypoxic tumor microenvironment (TME) [141]. Given that most solid tumors exhibit a pH from 6.5 to 7.4, milk exosomes are conjugated to doxorubicin by a pH-sensitive imine bond, which gets cleaved and releases doxorubicin in an acidic TME. Furthermore, photodynamic therapy is introduced by encapsulating photosensitizer-chlorin e6 (Ce6) and the anthracene endoperoxide derivative (EPT1) within milk exosomes. Upon exposure to 808 nm near-infrared light, Ce6 releases plasmonic heat that generates singlet oxygen from EPT1 and corrects hypoxia in TME. This Exo@Dox-EPT1 nanocarrier shows enhanced antitumor effects and biocompatibility with less cardiotoxicity caused by doxorubicin in an in vitro and in vivo mouse model of oral squamous cell carcinoma.

## 6. Conclusions

With the significant advancements in technology analyzing nucleic acids, proteins, and microbiota, HBM and animal-derived milk are now known to contain distinct bioactive molecules along with renowned nutritional components, and their therapeutic roles are becoming appreciated. In particular, milk-derived exosomes have gathered much attention owing to their intrinsic antitumor activities. The milk exosomes modulate immune function and suppress the proliferation of various cancer cells in vitro and in vivo, and their chemotherapeutic function is mainly due to the exosomal miRNAs with immunoregulatory and tumor-suppressive activities. The milk exosomes can also serve as oral delivery vehicles for chemotherapeutic agents and siRNAs, and further modification of the exosomes with a tumor-targeting ligand enables the targeted delivery of drugs to the tumor sites.

Given the physiological activity, safety, biocompatibility, and drug delivery potential, the application of milk exosomes in cancer therapeutics are innumerable. Future studies are required to demonstrate how individual bioactive components within exosomes exert biological function, including antitumor activity, and to clarify any possible unwanted effects upon exosome treatment. In addition, it would be important to establish a cost effective and standardized method to isolate, purify, and manipulate exosomes from milk to ensure the quality of the exosomes for clinical and industrial implementation. 

## Figures and Tables

**Table 1:** Immune-regulating miRNAs in milk.

miRNA	Immune Function	Reference
miR-17 and miR-92 cluster	B-cell, T-cell, and monocyte development	[108]
miR-29a-3p	Suppression of immune responses to intracellular pathogens by targeting IFN-γ	[99]
miR-30b-5p	Promotion of cellular invasion by directly targeting GalNAc transferase, immunosuppression by increasing IL-10	[99,111]
miR-106	Regulation of IL-10 production	[113]
miR-125b	Negative regulation of TNF-α production, activation, and sensitivity	[108]
miR-146b-5p	Negative regulation of the innate immune response by targeting NF-κB signaling, control of TLR and cytokine signaling	[108,109]
miR-150	Control of B cell differentiation, pre- and pro-B cell formation or function	[109]
miR-155	T- and B-cell maturation, the innate immune response	[108,109]
miR-181a, miR-181b	B-cell differentiation, CD4^+^ T-cell sensitivity and selection	[108,109,113]
miR-182-5p	Promotion of helper T cell-mediated immune responses upon induction by IL-2	[99,112]
miR-223	Neutrophil proliferation and activation	[108,109]
miR-451	Regulation of Macrophage migration inhibitory factor (MIF)	[113]
let-7i	Toll-like receptor 4 expression in human cholangiocytes	[108]

IFN-γ, interferon- γ; GalNAc, N-acetylgalactosamine; IL, interleukin; NF-κB, nuclear factor kappa-light-chain-enhancer of activated B cells.

## Abbreviations

HBM	human breast milk
NEC	necrotizing enterocolitis
HMOs	human milk oligosaccharides
EVs	extracellular vesicles
TGF-β	transforming growth factor β
TME	tumor microenvironment

## References

[1] Section on Breastfeeding (2012). Breastfeeding and the Use of Human Milk. Pediatrics.

[2] Gartner L.M., Morton J., Lawrence R.A., Naylor A.J., O’Hare D., Schanler R.J., Eidelman A.I. (2005). Breastfeeding and the use of human milk. Pediatrics.

[3] Agostoni C., Braegger C., Decsi T., Kolacek S., Koletzko B., Michaelsen K.F., Mihatsch W., Moreno L.A., Puntis J., Shamir R. (2009). Breast-feeding: A commentary by the ESPGHAN Committee on Nutrition. J. Pediatr. Gastroenterol. Nutr..

[4] Bernard J.Y., De Agostini M., Forhan A., Alfaiate T., Bonet M., Champion V., Kaminski M., de Lauzon-Guillain B., Charles M.A., Heude B. (2013). Breastfeeding duration and cognitive development at 2 and 3 years of age in the EDEN mother-child cohort. J. Pediatr..

[5] van Rossum C.T.M., Buchner F.L., Hoekstra J. (2006). Quantification of Health Effects of Breastfeeding—Review of the Literature and Model Simulation.

[6] Ip S., Chung M., Raman G., Chew P., Magula N., DeVine D., Trikalinos T., Lau J. (2007). Breastfeeding and maternal and infant health outcomes in developed countries. Evid Rep. Technol. Assess. (Full Rep.).

[7] Güngör D., Nadaud P., Dreibelbis C., LaPergola C.C., Wong Y.P., Terry N., Abrams S.A., Beker L., Jacobovits T., Järvinen K.M. (2019). Infant milk-feeding practices and childhood leukemia: A systematic review. Am. J. Clin. Nutr..

[8] Koletzko B., Baker S., Cleghorn G., Neto U.F., Gopalan S., Hernell O., Hock Q.S., Jirapinyo P., Lonnerdal B., Pencharz P. (2005). Global standard for the composition of infant formula: Recommendations of an ESPGHAN coordinated international expert group. J. Pediatr. Gastroenterol. Nutr..

[9] Kim M.H., Shim K.S., Yi D.Y., Lim I.S., Chae S.A., Yun S.W., Lee N.M., Kim S.Y., Kim S. (2019). Macronutrient Analysis of Human Milk according to Storage and Processing in Korean Mother. Pediatr. Gastroenterol. Hepatol. Nutr..

[10] Ballard O., Morrow A.L. (2013). Human milk composition: Nutrients and bioactive factors. Pediatr. Clin. N. Am..

[11] Kim S.Y., Yi D.Y. (2020). Components of human breast milk: From macronutrient to microbiome and microRNA. Clin. Exp. Pediatr..

[12] Kim S.Y., Yi D.Y. (2020). Analysis of the human breast milk microbiome and bacterial extracellular vesicles in healthy mothers. Exp. Mol. Med..

[13] Hegar B., Wibowo Y., Basrowi R.W., Ranuh R.G., Sudarmo S.M., Munasir Z., Widodo A.D., Kadim M., Suryawan A., Diana N.R. (2019). The Role of Two Human Milk Oligosaccharides, 2’-Fucosyllactose and Lacto-N-Neotetraose, in Infant Nutrition. Pediatr. Gastroenterol. Hepatol. Nutr..

[14] Walker A.W., Ince J., Duncan S.H., Webster L.M., Holtrop G., Ze X., Brown D., Stares M.D., Scott P., Bergerat A. (2011). Dominant and diet-responsive groups of bacteria within the human colonic microbiota. ISME J..

[15] Plaza-Díaz J., Fontana L., Gil A. (2018). Human Milk Oligosaccharides and Immune System Development. Nutrients.

[16] Lönnerdal B., Woodhouse L.R., Glazier C. (1987). Compartmentalization and quantitation of protein in human milk. J. Nutr..

[17] Lönnerdal B., Lien E.L. (2003). Nutritional and physiologic significance of alpha-lactalbumin in infants. Nutr. Rev..

[18] Martin C.R., Ling P.R., Blackburn G.L. (2016). Review of Infant Feeding: Key Features of Breast Milk and Infant Formula. Nutrients.

[19] Perrine C.G., Sharma A.J., Jefferds M.E., Serdula M.K., Scanlon K.S. (2010). Adherence to vitamin D recommendations among US infants. Pediatrics.

[20] Committee on Fetus and Newborn (2003). Controversies concerning vitamin K and the newborn. American Academy of Pediatrics Committee on Fetus and Newborn. Pediatrics.

[21] Hill D.R., Newburg D.S. (2015). Clinical applications of bioactive milk components. Nutr. Rev..

[22] Parodi P.W. (2007). A role for milk proteins and their peptides in cancer prevention. Curr. Pharm. Des..

[23] Kozu T., Iinuma G., Ohashi Y., Saito Y., Akasu T., Saito D., Alexander D.B., Iigo M., Kakizoe T., Tsuda H. (2009). Effect of orally administered bovine lactoferrin on the growth of adenomatous colorectal polyps in a randomized, placebo-controlled clinical trial. Cancer Prev. Res..

[24] Parikh P.M., Vaid A., Advani S.H., Digumarti R., Madhavan J., Nag S., Bapna A., Sekhon J.S., Patil S., Ismail P.M. (2011). Randomized, double-blind, placebo-controlled phase II study of single-agent oral talactoferrin in patients with locally advanced or metastatic non-small-cell lung cancer that progressed after chemotherapy. J. Clin. Oncol..

[25] Digumarti R., Wang Y., Raman G., Doval D.C., Advani S.H., Julka P.K., Parikh P.M., Patil S., Nag S., Madhavan J. (2011). A randomized, double-blind, placebo-controlled, phase II study of oral talactoferrin in combination with carboplatin and paclitaxel in previously untreated locally advanced or metastatic non-small cell lung cancer. J. Thorac. Oncol..

[26] Sun X., Jiang R., Przepiorski A., Reddy S., Palmano K.P., Krissansen G.W. (2012). “Iron-saturated” bovine lactoferrin improves the chemotherapeutic effects of tamoxifen in the treatment of basal-like breast cancer in mice. BMC Cancer.

[27] Fischer W., Gustafsson L., Mossberg A.K., Gronli J., Mork S., Bjerkvig R., Svanborg C. (2004). Human alpha-lactalbumin made lethal to tumor cells (HAMLET) kills human glioblastoma cells in brain xenografts by an apoptosis-like mechanism and prolongs survival. Cancer Res..

[28] Mossberg A.K., Hun Mok K., Morozova-Roche L.A., Svanborg C. (2010). Structure and function of human α-lactalbumin made lethal to tumor cells (HAMLET)-type complexes. FEBS J..

[29] Gustafsson L., Aits S., Onnerfjord P., Trulsson M., Storm P., Svanborg C. (2009). Changes in proteasome structure and function caused by HAMLET in tumor cells. PLoS ONE.

[30] Heikkilä M.P., Saris P.E. (2003). Inhibition of Staphylococcus aureus by the commensal bacteria of human milk. J. Appl. Microbiol..

[31] Hunt K.M., Foster J.A., Forney L.J., Schütte U.M., Beck D.L., Abdo Z., Fox L.K., Williams J.E., McGuire M.K., McGuire M.A. (2011). Characterization of the diversity and temporal stability of bacterial communities in human milk. PLoS ONE.

[32] Fitzstevens J.L., Smith K.C., Hagadorn J.I., Caimano M.J., Matson A.P., Brownell E.A. (2017). Systematic Review of the Human Milk Microbiota. Nutr. Clin. Pract..

[33] Togo A., Dufour J.C., Lagier J.C., Dubourg G., Raoult D., Million M. (2019). Repertoire of human breast and milk microbiota: A systematic review. Future Microbiol..

[34] Ramsay D.T., Kent J.C., Owens R.A., Hartmann P.E. (2004). Ultrasound imaging of milk ejection in the breast of lactating women. Pediatrics.

[35] Civardi E., Garofoli F., Tzialla C., Paolillo P., Bollani L., Stronati M. (2013). Microorganisms in human milk: Lights and shadows. J. Matern. Fetal Neonatal. Med..

[36] Rodríguez J.M. (2014). The origin of human milk bacteria: Is there a bacterial entero-mammary pathway during late pregnancy and lactation?. Adv. Nutr..

[37] Gomez-Llorente C., Plaza-Diaz J., Aguilera M., Muñoz-Quezada S., Bermudez-Brito M., Peso-Echarri P., Martinez-Silla R., Vasallo-Morillas M.I., Campaña-Martin L., Vives-Piñera I. (2013). Three main factors define changes in fecal microbiota associated with feeding modality in infants. J. Pediatr. Gastroenterol. Nutr..

[38] Martín V., Maldonado-Barragán A., Moles L., Rodriguez-Baños M., Campo R.D., Fernández L., Rodríguez J.M., Jiménez E. (2012). Sharing of bacterial strains between breast milk and infant feces. J. Hum. Lact..

[39] Coppa G.V., Bruni S., Morelli L., Soldi S., Gabrielli O. (2004). The first prebiotics in humans: Human milk oligosaccharides. J. Clin. Gastroenterol..

[40] Oozeer R., van Limpt K., Ludwig T., Ben Amor K., Martin R., Wind R.D., Boehm G., Knol J. (2013). Intestinal microbiology in early life: Specific prebiotics can have similar functionalities as human-milk oligosaccharides. Am. J. Clin. Nutr..

[41] Zimmermann P., Curtis N. (2018). Factors Influencing the Intestinal Microbiome During the First Year of Life. Pediatr. Infect. Dis. J..

[42] Moossavi S., Sepehri S., Robertson B., Bode L., Goruk S., Field C.J., Lix L.M., de Souza R.J., Becker A.B., Mandhane P.J. (2019). Composition and Variation of the Human Milk Microbiota Are Influenced by Maternal and Early-Life Factors. Cell Host Microbe.

[43] Yoo J.Y., Rho M., You Y.A., Kwon E.J., Kim M.H., Kym S., Jee Y.K., Kim Y.K., Kim Y.J. (2016). 16S rRNA gene-based metagenomic analysis reveals differences in bacteria-derived extracellular vesicles in the urine of pregnant and non-pregnant women. Exp. Mol. Med..

[44] Rashed M.H., Bayraktar E., Helal G.K., Abd-Ellah M.F., Amero P., Chavez-Reyes A., Rodriguez-Aguayo C. (2017). Exosomes: From Garbage Bins to Promising Therapeutic Targets. Int. J. Mol. Sci..

[45] Hessvik N.P., Llorente A. (2018). Current knowledge on exosome biogenesis and release. Cell Mol. Life Sci..

[46] Vlassov A.V., Magdaleno S., Setterquist R., Conrad R. (2012). Exosomes: Current knowledge of their composition, biological functions, and diagnostic and therapeutic potentials. Biochim. Biophys. Acta.

[47] Admyre C., Johansson S.M., Qazi K.R., Filén J.J., Lahesmaa R., Norman M., Neve E.P., Scheynius A., Gabrielsson S. (2007). Exosomes with immune modulatory features are present in human breast milk. J. Immunol..

[48] Galley J.D., Besner G.E. (2020). The Therapeutic Potential of Breast Milk-Derived Extracellular Vesicles. Nutrients.

[49] Hata T., Murakami K., Nakatani H., Yamamoto Y., Matsuda T., Aoki N. (2010). Isolation of bovine milk-derived microvesicles carrying mRNAs and microRNAs. Biochem. Biophys. Res. Commun..

[50] Gu Y., Li M., Wang T., Liang Y., Zhong Z., Wang X., Zhou Q., Chen L., Lang Q., He Z. (2012). Lactation-related microRNA expression profiles of porcine breast milk exosomes. PLoS ONE.

[51] Chen Z., Xie Y., Luo J., Chen T., Xi Q., Zhang Y., Sun J. (2020). Milk exosome-derived miRNAs from water buffalo are implicated in immune response and metabolism process. BMC Vet. Res..

[52] Baddela V.S., Nayan V., Rani P., Onteru S.K., Singh D. (2016). Physicochemical Biomolecular Insights into Buffalo Milk-Derived Nanovesicles. Appl. Biochem. Biotechnol..

[53] Badawy A.A., El-Magd M.A., AlSadrah S.A. (2018). Therapeutic Effect of Camel Milk and Its Exosomes on MCF7 Cells In Vitro and In Vivo. Integr. Cancer Ther..

[54] Ma Y., Feng S., Wang X., Qazi I.H., Long K., Luo Y., Li G., Ning C., Wang Y., Hu S. (2018). Exploration of exosomal microRNA expression profiles in pigeon ‘Milk’ during the lactation period. BMC Genom..

[55] Quan S., Nan X., Wang K., Jiang L., Yao J., Xiong B. (2020). Characterization of Sheep Milk Extracellular Vesicle-miRNA by Sequencing and Comparison with Cow Milk. Animals.

[56] Gallier S., Vocking K., Post J.A., Van De Heijning B., Acton D., Van Der Beek E.M., Van Baalen T. (2015). A novel infant milk formula concept: Mimicking the human milk fat globule structure. Colloids Surf. B Biointerfaces.

[57] Torregrosa Paredes P., Gutzeit C., Johansson S., Admyre C., Stenius F., Alm J., Scheynius A., Gabrielsson S. (2014). Differences in exosome populations in human breast milk in relation to allergic sensitization and lifestyle. Allergy.

[58] Hock A., Miyake H., Li B., Lee C., Ermini L., Koike Y., Chen Y., Määttänen P., Zani A., Pierro A. (2017). Breast milk-derived exosomes promote intestinal epithelial cell growth. J. Pediatr. Surg..

[59] Maffei D., Schanler R.J. (2017). Human milk is the feeding strategy to prevent necrotizing enterocolitis!. Semin. Perinatol..

[60] Cortez J., Makker K., Kraemer D.F., Neu J., Sharma R., Hudak M.L. (2018). Maternal milk feedings reduce sepsis, necrotizing enterocolitis and improve outcomes of premature infants. J. Perinatol..

[61] O’Connor D.L., Gibbins S., Kiss A., Bando N., Brennan-Donnan J., Ng E., Campbell D.M., Vaz S., Fusch C., Asztalos E. (2016). Effect of Supplemental Donor Human Milk Compared with Preterm Formula on Neurodevelopment of Very Low-Birth-Weight Infants at 18 Months: A Randomized Clinical Trial. JAMA.

[62] Quigley M., Embleton N.D., McGuire W. (2018). Formula versus donor breast milk for feeding preterm or low birth weight infants. Cochrane Database Syst. Rev..

[63] Martin C., Patel M., Williams S., Arora H., Sims B. (2018). Human breast milk-derived exosomes attenuate cell death in intestinal epithelial cells. Innate Immun..

[64] Miyake H., Lee C., Chusilp S., Bhalla M., Li B., Pitino M., Seo S., O’Connor D.L., Pierro A. (2020). Human breast milk exosomes attenuate intestinal damage. Pediatr. Surg. Int..

[65] Wang X., Yan X., Zhang L., Cai J., Zhou Y., Liu H., Hu Y., Chen W., Xu S., Liu P. (2019). Identification and Peptidomic Profiling of Exosomes in Preterm Human Milk: Insights Into Necrotizing Enterocolitis Prevention. Mol. Nutr. Food Res..

[66] Li B., Hock A., Wu R.Y., Minich A., Botts S.R., Lee C., Antounians L., Miyake H., Koike Y., Chen Y. (2019). Bovine milk-derived exosomes enhance goblet cell activity and prevent the development of experimental necrotizing enterocolitis. PLoS ONE.

[67] Arntz O.J., Pieters B.C., Oliveira M.C., Broeren M.G., Bennink M.B., de Vries M., van Lent P.L., Koenders M.I., van den Berg W.B., van der Kraan P.M. (2015). Oral administration of bovine milk derived extracellular vesicles attenuates arthritis in two mouse models. Mol. Nutr. Food Res..

[68] Näslund T.I., Paquin-Proulx D., Paredes P.T., Vallhov H., Sandberg J.K., Gabrielsson S. (2014). Exosomes from breast milk inhibit HIV-1 infection of dendritic cells and subsequent viral transfer to CD4+ T cells. Aids.

[69] Komine-Aizawa S., Ito S., Aizawa S., Namiki T., Hayakawa S. (2020). Cow milk exosomes activate NK cells and γδT cells in human PBMCs in vitro. Immunol. Med..

[70] Matic S., D’Souza D.H., Wu T., Pangloli P., Dia V.P. (2020). Bovine Milk Exosomes Affect Proliferation and Protect Macrophages against Cisplatin-Induced Cytotoxicity. Immunol. Investig..

[71] Samuel M., Chisanga D., Liem M., Keerthikumar S., Anand S., Ang C.S., Adda C.G., Versteegen E., Jois M., Mathivanan S. (2017). Bovine milk-derived exosomes from colostrum are enriched with proteins implicated in immune response and growth. Sci. Rep..

[72] Reinhardt T.A., Lippolis J.D., Nonnecke B.J., Sacco R.E. (2012). Bovine milk exosome proteome. J. Proteomics.

[73] Amitay E., Keinan-Boker L. (2014). Breastfeeding and childhood leukemia and lymphoma. Harefuah.

[74] Amitay E.L., Dubnov Raz G., Keinan-Boker L. (2016). Breastfeeding, Other Early Life Exposures and Childhood Leukemia and Lymphoma. Nutr. Cancer.

[75] Küçükçongar A., Oğuz A., Pınarlı F.G., Karadeniz C., Okur A., Kaya Z., Çelik B. (2015). Breastfeeding and Childhood Cancer: Is Breastfeeding Preventative to Childhood Cancer?. Pediatr. Hematol. Oncol..

[76] Mathur G.P., Gupta N., Mathur S., Gupta V., Pradhan S., Dwivedi J.N., Tripathi B.N., Kushwaha K.P., Sathy N., Modi U.J. (1993). Breastfeeding and childhood cancer. Indian Pediatr..

[77] Shu X.O., Clemens J., Zheng W., Ying D.M., Ji B.T., Jin F. (1995). Infant breastfeeding and the risk of childhood lymphoma and leukaemia. Int. J. Epidemiol..

[78] Martin R.M., Gunnell D., Owen C.G., Smith G.D. (2005). Breast-feeding and childhood cancer: A systematic review with metaanalysis. Int. J. Cancer.

[79] Amitay E.L., Keinan-Boker L. (2015). Breastfeeding and Childhood Leukemia Incidence: A Meta-analysis and Systematic Review. JAMA Pediatr..

[80] Reif S., Elbaum Shiff Y., Golan-Gerstl R. (2019). Milk-derived exosomes (MDEs) have a different biological effect on normal fetal colon epithelial cells compared to colon tumor cells in a miRNA-dependent manner. J. Transl. Med..

[81] Munagala R., Aqil F., Jeyabalan J., Agrawal A.K., Mudd A.M., Kyakulaga A.H., Singh I.P., Vadhanam M.V., Gupta R.C. (2017). Exosomal formulation of anthocyanidins against multiple cancer types. Cancer Lett..

[82] Korashy H.M., El Gendy M.A., Alhaider A.A., El-Kadi A.O. (2012). Camel milk modulates the expression of aryl hydrocarbon receptor-regulated genes, Cyp1a1, Nqo1, and Gsta1, in murine hepatoma Hepa 1c1c7 cells. J. Biomed. Biotechnol..

[83] Korashy H.M., Maayah Z.H., Abd-Allah A.R., El-Kadi A.O., Alhaider A.A. (2012). Camel milk triggers apoptotic signaling pathways in human hepatoma HepG2 and breast cancer MCF7 cell lines through transcriptional mechanism. J. Biomed. Biotechnol..

[84] Qin W., Tsukasaki Y., Dasgupta S., Mukhopadhyay N., Ikebe M., Sauter E.R. (2016). Exosomes in Human Breast Milk Promote EMT. Clin. Cancer Res..

[85] Bartel D.P., Chen C.Z. (2004). Micromanagers of gene expression: The potentially widespread influence of metazoan microRNAs. Nat. Rev. Genet..

[86] Bartel D.P. (2004). MicroRNAs: Genomics, biogenesis, mechanism, and function. Cell.

[87] Friedman R.C., Farh K.K., Burge C.B., Bartel D.P. (2009). Most mammalian mRNAs are conserved targets of microRNAs. Genome Res..

[88] Ambros V. (2003). MicroRNA pathways in flies and worms: Growth, death, fat, stress, and timing. Cell.

[89] Carrington J.C., Ambros V. (2003). Role of microRNAs in plant and animal development. Science.

[90] Chang T.C., Mendell J.T. (2007). microRNAs in vertebrate physiology and human disease. Annu. Rev. Genom. Hum. Genet..

[91] Bushati N., Cohen S.M. (2007). microRNA functions. Annu. Rev. Cell Dev. Biol..

[92] Mollaei H., Safaralizadeh R., Rostami Z. (2019). MicroRNA replacement therapy in cancer. J. Cell Physiol..

[93] Rupaimoole R., Slack F.J. (2017). MicroRNA therapeutics: Towards a new era for the management of cancer and other diseases. Nat. Rev. Drug Discov..

[94] Garzon R., Calin G.A., Croce C.M. (2009). MicroRNAs in Cancer. Annu. Rev. Med..

[95] Ling H., Fabbri M., Calin G.A. (2013). MicroRNAs and other non-coding RNAs as targets for anticancer drug development. Nat. Rev. Drug Discov..

[96] Alsaweed M., Hepworth A.R., Lefèvre C., Hartmann P.E., Geddes D.T., Hassiotou F. (2015). Human Milk MicroRNA and Total RNA Differ Depending on Milk Fractionation. J. Cell Biochem..

[97] Alsaweed M., Lai C.T., Hartmann P.E., Geddes D.T., Kakulas F. (2016). Human milk miRNAs primarily originate from the mammary gland resulting in unique miRNA profiles of fractionated milk. Sci. Rep..

[98] Alsaweed M., Hartmann P.E., Geddes D.T., Kakulas F. (2015). MicroRNAs in Breastmilk and the Lactating Breast: Potential Immunoprotectors and Developmental Regulators for the Infant and the Mother. Int. J. Environ. Res. Public Health.

[99] Zhou Q., Li M., Wang X., Li Q., Wang T., Zhu Q., Zhou X., Gao X., Li X. (2012). Immune-related microRNAs are abundant in breast milk exosomes. Int. J. Biol. Sci..

[100] Munch E.M., Harris R.A., Mohammad M., Benham A.L., Pejerrey S.M., Showalter L., Hu M., Shope C.D., Maningat P.D., Gunaratne P.H. (2013). Transcriptome profiling of microRNA by Next-Gen deep sequencing reveals known and novel miRNA species in the lipid fraction of human breast milk. PLoS ONE.

[101] Lönnerdal B. (2019). Human Milk MicroRNAs/Exosomes: Composition and Biological Effects. Nestle Nutr. Inst. Workshop Ser..

[102] Izumi H., Kosaka N., Shimizu T., Sekine K., Ochiya T., Takase M. (2012). Bovine milk contains microRNA and messenger RNA that are stable under degradative conditions. J. Dairy Sci..

[103] Liao Y., Du X., Li J., Lönnerdal B. (2017). Human milk exosomes and their microRNAs survive digestion in vitro and are taken up by human intestinal cells. Mol. Nutr. Food Res..

[104] Kahn S., Liao Y., Du X., Xu W., Li J., Lönnerdal B. (2018). Exosomal MicroRNAs in Milk from Mothers Delivering Preterm Infants Survive in Vitro Digestion and Are Taken Up by Human Intestinal Cells. Mol. Nutr. Food Res..

[105] Zempleni J., Aguilar-Lozano A., Sadri M., Sukreet S., Manca S., Wu D., Zhou F., Mutai E. (2017). Biological Activities of Extracellular Vesicles and Their Cargos from Bovine and Human Milk in Humans and Implications for Infants. J. Nutr..

[106] Golan-Gerstl R., Elbaum Shiff Y., Moshayoff V., Schecter D., Leshkowitz D., Reif S. (2017). Characterization and biological function of milk-derived miRNAs. Mol. Nutr. Food Res..

[107] Nojiri K., Kobayashi S., Higurashi S., Takahashi T., Tsujimori Y., Ueno H.M., Watanabe-Matsuhashi S., Toba Y., Yamamura J., Nakano T. (2020). Maternal Health and Nutrition Status, Human Milk Composition, and Growth and Development of Infants and Children: A Prospective Japanese Human Milk Study Protocol. Int. J. Environ. Res. Public Health.

[108] Kosaka N., Izumi H., Sekine K., Ochiya T. (2010). microRNA as a new immune-regulatory agent in breast milk. Silence.

[109] Na R.S., E G.X., Sun W., Sun X.W., Qiu X.Y., Chen L.P., Huang Y.F. (2015). Expressional analysis of immune-related miRNAs in breast milk. Genet. Mol. Res..

[110] Chen X., Gao C., Li H., Huang L., Sun Q., Dong Y., Tian C., Gao S., Dong H., Guan D. (2010). Identification and characterization of microRNAs in raw milk during different periods of lactation, commercial fluid, and powdered milk products. Cell Res..

[111] Gaziel-Sovran A., Segura M.F., Di Micco R., Collins M.K., Hanniford D., Vega-Saenz de Miera E., Rakus J.F., Dankert J.F., Shang S., Kerbel R.S. (2011). miR-30b/30d regulation of GalNAc transferases enhances invasion and immunosuppression during metastasis. Cancer Cell.

[112] Stittrich A.B., Haftmann C., Sgouroudis E., Kühl A.A., Hegazy A.N., Panse I., Riedel R., Flossdorf M., Dong J., Fuhrmann F. (2010). The microRNA miR-182 is induced by IL-2 and promotes clonal expansion of activated helper T lymphocytes. Nat. Immunol..

[113] Sun Q., Chen X., Yu J., Zen K., Zhang C.Y., Li L. (2013). Immune modulatory function of abundant immune-related microRNAs in microvesicles from bovine colostrum. Protein Cell.

[114] Melnik B.C., Schmitz G. (2019). Exosomes of pasteurized milk: Potential pathogens of Western diseases. J. Transl. Med..

[115] Long X.R., He Y., Huang C., Li J. (2014). MicroRNA-148a is silenced by hypermethylation and interacts with DNA methyltransferase 1 in hepatocellular carcinogenesis. Int. J. Oncol..

[116] Li J., Song Y., Wang Y., Luo J., Yu W. (2013). MicroRNA-148a suppresses epithelial-to-mesenchymal transition by targeting ROCK1 in non-small cell lung cancer cells. Mol. Cell Biochem..

[117] Yu J., Li Q., Xu Q., Liu L., Jiang B. (2011). MiR-148a inhibits angiogenesis by targeting ERBB3. J. Biomed. Res..

[118] Zhang H., Luo X.Q., Zhang P., Huang L.B., Zheng Y.S., Wu J., Zhou H., Qu L.H., Xu L., Chen Y.Q. (2009). MicroRNA patterns associated with clinical prognostic parameters and CNS relapse prediction in pediatric acute leukemia. PLoS ONE.

[119] de Oliveira J.C., Brassesco M.S., Scrideli C.A., Tone L.G., Narendran A. (2012). MicroRNA expression and activity in pediatric acute lymphoblastic leukemia (ALL). Pediatr. Blood Cancer.

[120] Tomé-Carneiro J., Fernández-Alonso N., Tomás-Zapico C., Visioli F., Iglesias-Gutierrez E., Dávalos A. (2018). Breast milk microRNAs harsh journey towards potential effects in infant development and maturation. Lipid encapsulation can help. Pharmacol. Res..

[121] Melnik B.C. (2015). MiR-21: An environmental driver of malignant melanoma?. J. Transl. Med..

[122] Meng F., Henson R., Wehbe-Janek H., Ghoshal K., Jacob S.T., Patel T. (2007). MicroRNA-21 regulates expression of the PTEN tumor suppressor gene in human hepatocellular cancer. Gastroenterology.

[123] Sayed D., Rane S., Lypowy J., He M., Chen I.Y., Vashistha H., Yan L., Malhotra A., Vatner D., Abdellatif M. (2008). MicroRNA-21 targets Sprouty2 and promotes cellular outgrowths. Mol. Biol. Cell.

[124] Asangani I.A., Rasheed S.A., Nikolova D.A., Leupold J.H., Colburn N.H., Post S., Allgayer H. (2008). MicroRNA-21 (miR-21) post-transcriptionally downregulates tumor suppressor Pdcd4 and stimulates invasion, intravasation and metastasis in colorectal cancer. Oncogene.

[125] Cao Q., Li Y.Y., He W.F., Zhang Z.Z., Zhou Q., Liu X., Shen Y., Huang T.T. (2013). Interplay between microRNAs and the STAT3 signaling pathway in human cancers. Physiol. Genom..

[126] Huang C., Li H., Wu W., Jiang T., Qiu Z. (2013). Regulation of miR-155 affects pancreatic cancer cell invasiveness and migration by modulating the STAT3 signaling pathway through SOCS1. Oncol. Rep..

[127] Ha D., Yang N., Nadithe V. (2016). Exosomes as therapeutic drug carriers and delivery vehicles across biological membranes: Current perspectives and future challenges. Acta Pharm. Sin. B.

[128] Munagala R., Aqil F., Jeyabalan J., Gupta R.C. (2016). Bovine milk-derived exosomes for drug delivery. Cancer Lett..

[129] Aqil F., Munagala R., Jeyabalan J., Agrawal A.K., Kyakulaga A.H., Wilcher S.A., Gupta R.C. (2019). Milk exosomes—Natural nanoparticles for siRNA delivery. Cancer Lett..

[130] Betker J.L., Angle B.M., Graner M.W., Anchordoquy T.J. (2019). The Potential of Exosomes from Cow Milk for Oral Delivery. J. Pharm. Sci..

[131] Carobolante G., Mantaj J., Ferrari E., Vllasaliu D. (2020). Cow Milk and Intestinal Epithelial Cell-derived Extracellular Vesicles as Systems for Enhancing Oral Drug Delivery. Pharmaceutics.

[132] Aqil F., Kausar H., Agrawal A.K., Jeyabalan J., Kyakulaga A.H., Munagala R., Gupta R. (2016). Exosomal formulation enhances therapeutic response of celastrol against lung cancer. Exp. Mol. Pathol..

[133] Agrawal A.K., Aqil F., Jeyabalan J., Spencer W.A., Beck J., Gachuki B.W., Alhakeem S.S., Oben K., Munagala R., Bondada S. (2017). Milk-derived exosomes for oral delivery of paclitaxel. Nanomedicine.

[134] Dong Y., Siegwart D.J., Anderson D.G. (2019). Strategies, design, and chemistry in siRNA delivery systems. Adv. Drug Deliv. Rev..

[135] Das M., Musetti S., Huang L. (2019). RNA Interference-Based Cancer Drugs: The Roadblocks, and the “Delivery” of the Promise. Nucleic Acid Ther..

[136] Tao H., Xu H., Zuo L., Li C., Qiao G., Guo M., Zheng L., Leitgeb M., Lin X. (2020). Exosomes-coated bcl-2 siRNA inhibits the growth of digestive system tumors both in vitro and in vivo. Int. J. Biol. Macromol..

[137] Naor D., Nedvetzki S., Golan I., Melnik L., Faitelson Y. (2002). CD44 in cancer. Crit. Rev. Clin. Lab. Sci..

[138] Li D., Yao S., Zhou Z., Shi J., Huang Z., Wu Z. (2020). Hyaluronan decoration of milk exosomes directs tumor-specific delivery of doxorubicin. Carbohydr. Res..

[139] Sugahara K.N., Teesalu T., Karmali P.P., Kotamraju V.R., Agemy L., Greenwald D.R., Ruoslahti E. (2010). Coadministration of a tumor-penetrating peptide enhances the efficacy of cancer drugs. Science.

[140] Teesalu T., Sugahara K.N., Kotamraju V.R., Ruoslahti E. (2009). C-end rule peptides mediate neuropilin-1-dependent cell, vascular, and tissue penetration. Proc. Natl. Acad. Sci. USA.

[141] Zhang Q., Xiao Q., Yin H., Xia C., Pu Y., He Z., Hu Q., Wang J., Wang Y. (2020). Milk-exosome based pH/light sensitive drug system to enhance anticancer activity against oral squamous cell carcinoma. RSC Adv..

